# Chronic kidney disease is associated with a higher 90-day mortality than other chronic medical conditions in patients with sepsis

**DOI:** 10.1038/srep10539

**Published:** 2015-05-21

**Authors:** Ashham Mansur, Evelyn Mulwande, Maximilian Steinau, Ingo Bergmann, Aron Frederik Popov, Michael Ghadimi, Tim Beissbarth, Martin Bauer, José Hinz

**Affiliations:** 1 Department of Anesthesiology, University Medical Center, Georg August University, Robert-Koch-Str.40, D-37075 Goettingen, Germany; 2 Department of Cardiothoracic Transplantation & Mechanical Support, Royal Brompton and Harefield Hospital, Harefield, Hill End Road, UB9 6JH London, United Kingdom; 3 Department of General and Visceral Surgery, University Medical Center, Georg August University, D-37075 Goettingen, Germany; 4 Department of Medical Statistics, University Medical Center, Georg August University, Robert-Koch-Str.40, D-37075 Goettingen, Germany

## Abstract

According to previous studies, the clinical course of sepsis could be affected by preexisting medical conditions, which are very common among patients with sepsis. This observational study aimed at investigating whether common chronic medical conditions affect the 90-day mortality risk in adult Caucasian patients with sepsis. A total of 482 patients with sepsis were enrolled in this study. The ninety-day mortality was the primary outcome; organ failure was the secondary outcome. Sepsis-related organ failure assessment (SOFA) scores and the requirements for organ support were evaluated to assess organ failure. A multivariate Cox regression model for the association between the 90-day mortality risk and chronic preexisting medical conditions adjusted for all relevant confounders and mortality predictors revealed the highest hazard ratio for patients with chronic kidney disease (CKD) (hazard ratio, 2.25; 95% CI, 1.46-3.46; p = 0.0002). Patients with CKD had higher SOFA scores than patients without CKD (8.9 ± 4.0 and 6.5 ± 3.4, respectively; p < 0.0001). Additionally, an analysis of organ-specific SOFA scores revealed higher scores in three organ systems (kidney, cardiovascular and coagulation). Patients with CKD have the highest 90-day mortality risk compared with patients without CKD or with other chronic medical conditions.

Sepsis is a life-threatening complication of an infection accompanied by a systemic inflammatory response that might cause organ injury, shock and death^1^. In the United States, half of patients with severe sepsis are treated in the intensive care unit (ICU), and sepsis accounts for 10% of all ICU admissions^2^,^3^. The annual incidence of sepsis in the United States is estimated at 750,000 cases, and these numbers show a rising trend^4^.

The clinical course of sepsis could be affected by several factors. One of the most relevant factors is the presence of preexisting medical conditions, which are very common among patients with sepsis^5^,^6^. Previous studies have shown that medical comorbidities are associated with the severity of sepsis and the degree of organ dysfunction^7^,^8^. However, the majority of previous investigations have focused on individual chronic medical conditions^9^,^10^,^11^, and most studies that examined several chronic medical conditions were retrospective in nature^12^,^13^. Additionally, the major outcome of previous investigations was short-term mortality (28-day, ICU and hospital mortality)^12^,^13^,^14^,^15^. Because many sepsis patients remain hospitalized at day 28, and because strong evidence suggests that many late sequelae from sepsis are not captured by this time point^16^, many experts suggest that sepsis studies consider a larger window of time, such as 60 or 90 days^17^.

Moreover, as several improved treatments for sepsis, especially for sepsis-associated organ dysfunction, have been developed in recent years^18^, reevaluating the effect of common chronic medical conditions on the clinical course of patients with sepsis is important.

This prospective observational study aimed at investigating whether and to what extent common chronic medical conditions (arterial hypertension, coronary heart disease, chronic obstructive pulmonary disease (COPD), chronic kidney disease (CKD), insulin-dependent diabetes mellitus (IDDM), non-insulin-dependent diabetes mellitus (NIDDM), chronic liver disease, and history of stroke) affect the 90-day mortality risk in Caucasian patients with sepsis.

## Results

### Baseline characteristics

A total of 482 adult Caucasian patients with sepsis were enrolled in this observational investigation. All of the patients were successfully followed for a maximum of 90 days. The patients’ ages ranged from 19 to 92 years (median, 64 years) (Table 1); 35% were women and 65% were men. The distribution of sepsis/severe sepsis and septic shock was 40% and 60%, respectively. An assessment of the frequency of the eight chronic medical conditions revealed arterial hypertension to be the most common preexisting disease (55%) and history of stroke to be the least common (6%). Thirty percent of patients had a history of recent elective surgery, and 52% had a history of recent emergency surgery. The most common site of infection was the lung (55%).

At baseline, the mean sepsis-related organ failure assessment (SOFA) and acute physiology and chronic health evaluation II (APACHE II) morbidity scores were 9.1 ± 4.0 and 21.3 ± 7.0, respectively (Table 1). The frequency of organ support therapy (mechanical ventilation, vasopressor therapy and renal replacement therapy) at the time of sepsis onset was 84%, 60% and 9%, respectively (Table 1).

### Outcomes

#### Mortality

A multivariate Cox regression model for the association between the 90-day mortality risk and chronic preexisting medical conditions adjusted for all of the relevant confounders and mortality predictors revealed the highest hazard ratio for patients with CKD (hazard ratio, 2.25; 95% CI, 1.46-3.46; p = 0.0002) (Table 2) followed by those with diabetes mellitus (NIDDM: hazard ratio, 1.65; 95% CI, 0.96-2.83; p = 0.0684 and IDDM: hazard ratio, 1.62; 95% CI, 0.99-2.64; p = 0.0527) and a history of cancer (hazard ratio, 1.63; 95% CI, 1.09-2.34; p = 0.0182) (Table 2). This finding indicates that despite potential baseline confounders (age, gender, initial APACHE II and SOFA scores, septic shock, type of infection, recent surgical history), pre-existing CKD is an independent and significant prognostic variable for the 90-day mortality risk (Table 2).

**Figure 1:**
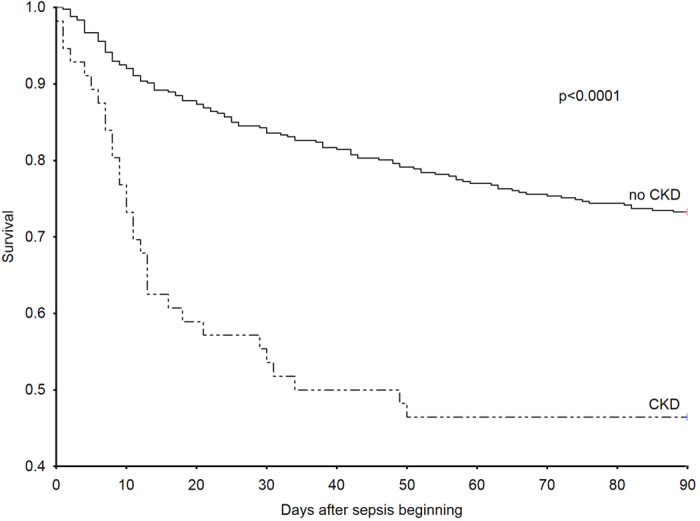
The Kaplan–Meier curves demonstrating survival were censored at day 90 for patients with CKD and patients without CKD. The mortality risk among the patients in the study was higher in the patients with CKD compared with the patients without CKD (p < 0.0001, log-rank test).

Similarly, a Kaplan-Meier survival analysis of the 90-day mortality risk among patients with CKD and patients without CKD revealed a significantly higher mortality risk among patients with CKD compared with patients without CKD (p < 0.0001, log-rank test) (Fig. 1). Similarly, patients with CKD had a significantly higher 28-day mortality rate compared to patients with no history of CKD (Table 3).

#### Disease severity

Over the 28-day observational period in the ICU, the mean SOFA score was 6.8 ± 3.6 (Table 3). Analyses of organ-specific SOFA scores revealed the highest scores in the respiratory, cardiovascular and central nervous systems (1.9 ± 0.8, 1.5 ± 1.0 and 1.9 ± 1.1, respectively).

To explore the effect of CKD (as the most predictive variable for mortality) on disease severity and the extent of organ dysfunction over the course of the ICU stay, we calculated organ-specific SOFA scores. Patients with CKD had higher SOFA scores compared with patients without CKD (8.9 ± 4.0 and 6.5 ± 3.4, respectively; p < 0.0001) (Table 3). Additionally, analyses of organ-specific SOFA scores revealed higher scores in three organ systems (kidney, cardiovascular and coagulation). Compared with patients without CKD, patients with CKD had higher SOFA-Renal scores (1.8 ± 1.4 and 0.7 ± 1.7, respectively; p < 0.0001), SOFA-Cardiovascular scores (1.9 ± 1.1 and 1.4 ± 0.9, respectively; p = 0.0012) and SOFA-Coagulation scores (0.6 ± 0.8 and 0.3 ± 0.5, respectively; p = 0.0242). The remaining two SOFA scores (respiratory and central nervous system) did not differ between the groups (Table 3).

Regarding organ support-free days, patients with CKD had significantly fewer vasopressor-free days compared with patients without CKD (7 ± 6 and 11 ± 7, respectively; p = 0.0002). CKD patients also had significantly fewer dialysis-free days compared with patients with no CKD history (11 ± 8 and 14 ± 8, respectively; p = 0.0030). The groups did not differ with regard to ventilator-free days.

## Discussion

This observational clinical investigation assessed the effect of the most common chronic medical conditions on the 90-day survival among patients with sepsis. According to our main findings, patients with CKD had the highest 90-day mortality risk compared with patients without CKD or patients with other chronic medical conditions. This observation underscores the negative consequences of pre-existing CKD for the clinical course of sepsis and confirms previous investigations on the role of CKD in sepsis^19^,^20^,^21^. Efforts are needed to reduce the incidence and control the negative effects of infections in patients with CKD.

The higher mortality risk among patients with CKD could be attributed to several factors, including uremia in patients with CKD. Uremia contributes to leukocyte dysfunction (lymphocyte, monocyte, neutrophil, and dendritic cells)^22^,^23^,^24^,^25^,^26^. Additionally, due to decreased renal clearance, patients with CKD are more likely to exhibit accumulations of inflammatory cytokines, which attenuate immune function^27^,^28^,^29^,^30^.

A major advantage of our study is the fact that we investigated, for the first time, organ-specific dysfunctions over the clinical course of disease with regard to CKD using organ-specific SOFA scores. Patients with CKD showed three higher organ-specific SOFA scores compared with patients without CKD. This result indicates more pronounced organ dysfunction in these three organ systems (cardiovascular, renal and coagulation). The higher SOFA-Cardiovascular score and the more frequent use of vasopressor therapy in patients with CKD compared with patients without CKD are consistent with previous observations demonstrating a higher demand for vasopressor administration in septic patients with renal failure^31^. The higher SOFA-Renal scores and the more frequent renal-replacement therapy in CKD patients compared with patients without CKD are intuitive, because sepsis patients with CKD are more likely to develop renal failure of acute or chronic kidney disease^2^,^19^,^31^,^32^. The observed higher SOFA-Coagulation scores, indicating severe thrombocytopenia, in the CKD group compared with the non-CKD group is in agreement with previous investigations showing that renal disease is associated with platelet dysfunction and thrombocytopenia^33^. Additionally, the pronounced thrombocytopenia observed in this patient group is consistent with the higher recorded mortality risk in these patients, because thrombocytopenia was shown to be a prognostic variable for mortality in patients with sepsis^7^.

The observed high mortality risk among patients with diabetes mellitus is in accordance with several previous studies showing that DM is associated with higher mortality caused by sepsis in several populations^11^. These findings are consistent with the fact that several aspects of immunity and host defense are altered in patients with DM^34^. Similarly, the high risk of mortality among patients with a history of cancer in our cohort is consistent with the results from previous studies^9^ and could be attributed to the fact that patients with cancer are at a high risk for developing a state of immunosuppression resulting from cancer therapy or the malignancy itself, thus leading to severe infection and sepsis, which is a is major cause of mortality in this group^35^,^36^.

Our study confirms the prognostic value of the initial morbidity scores, SOFA and APACHE II^37^. Furthermore, the significant association between age over 65 years and higher mortality risk is plausible.

Our study provides an important update of the prognostic value of the most common chronic medical conditions on the 90-day mortality risk among patients with sepsis. We found the highest mortality risk among patients with CKD, and much effort must be made to minimize the mortality risk in this group. Researchers and clinicians need to develop new treatment strategies, both preventive and curative, that are specially adapted for this patient group.

## Methods

### Patients

Adult Caucasian patients admitted to the surgical ICUs at the University Medical Center Goettingen (UMG) between April 2012 and July 2014 were screened daily according to the American College of Chest Physicians/Society of Critical Care Medicine (ACCP/SCCM) criteria for sepsis, severe sepsis, or septic shock^1^,^38^. Because interracial genetic differences might affect the clinical course of infectious diseases, we exclusively recruited Caucasians, who represent the majority of patients admitted to our surgical ICUs, into this prospective clinical investigation. Caucasian origin was assessed by questioning the patients, their next of kin or their legal representatives. This study conformed to the ethical principles of the Declaration of Helsinki (Seoul, 2008), and the study protocol was approved by the institutional ethics committee of the University of Goettingen in Goettingen, Germany. The study was performed in accordance with relevant guidelines and regulations. The methods were performed in accordance with the approved guidelines. Written informed consent was obtained either from the patients or from their legal representatives.

### Exclusion criteria

As described previously^39^, the exclusion criteria were: (1) age younger than 18; (2) pregnancy or breastfeeding (3) receipt of immunosuppressive therapy; (4) documented myocardial infarction within the previous 6 weeks; (5) New York Heart Association functional class IV chronic heart failure; (6) human immunodeficiency virus infection; (7) a do not resuscitate or do not treat order; (8) expected death within 28 days due to uncorrectable medical condition (e.g., poorly controlled neoplasm); (9) chronic vegetative state with pronounced neurological impairment; (10) current participation in any clinical trial (of a drug or device); (11) inability to be fully evaluated during the study period; and (12) study-site employee or a family member of a study-site employee.

### Data collection and clinical endpoints

Upon enrollment, the patient’s demographic characteristics, type of sepsis (sepsis/severe sepsis and septic shock), chronic comorbidities, recent surgical history (elective surgery, emergency surgery), site of infection and organ support were recorded. All of the patients were followed for 90 days, and the mortality risk within this observational period was recorded as the primary outcome. Sequential Organ Failure Assessment (SOFA)^40^ and Acute Physiology and Chronic Health Evaluation (APACHE) II^41^ scores were evaluated at the onset of sepsis. Organ dysfunction was reassessed over 28 days in the ICU using organ-specific SOFA scores to monitor morbidity. Organ failure (as assessed by SOFA scores), organ support requirements (mechanical ventilation, vasopressor therapy and renal-replacement therapy) and the length of ICU stay were recorded as secondary outcomes. Several relevant laboratory values were recorded as secondary variables. All of the relevant clinical data were obtained from the electronic patient record system (IntelliSpace Critical Care and Anesthesia (ICCA); Philips Healthcare, Andover, Massachusetts, USA); all medical records could be found in this system. Information regarding medical history and preexisting medical history were completed by examining previous physicians’ notes, through anamnestic questionnaires and by consulting each patient’s family doctor.

### Statistical analyses

The statistical analyses were performed using Statistica software (version 10; StatSoft, Tulsa, Oklahoma, USA). The significance of categorical variables was calculated using two-sided Fisher’s exact or chi-square tests, as appropriate. Two continuous variables were compared using the Mann-Whitney test. We performed a multivariate Cox regression analysis to examine the impact of common medical conditions on survival; several covariates, including mortality predictors (age, SOFA, APACHE II) and potential confounders (gender, BMI, septic shock, infection type, recent surgical history) were included in this model. Time-to-event data were compared using the log-rank test from the Statistica package for Kaplan-Meier survival analysis. A power calculation was performed using the Statistica package for power analysis. A p-value of  <0.05 was considered statistically significant.

## Additional Information

**How to cite this article**: Mansur, A. *et al.* Chronic kidney disease is associated with a higher 90-day mortality than other chronic medical conditions in patients with sepsis. *Sci. Rep.* **5**, 10539; doi: 10.1038/srep10539 (2015).

## Figures and Tables

**Table 1:** Patient baseline characteristics at the onset of sepsis.

	**All (n** = **482)**
Age [years]	63 ± 15
Gender, male, %	65
Body mass index	28 ± 7

Type of sepsis	
Sepsis/severe sepsis, %	40
Septic shock, %	60
SOFA score	9.1 ± 4.0
APACHE II score	21.3 ± 7.0

Comorbidities and recent surgical history, %	
Arterial hypertension	55
Coronary heart disease	7
Chronic obstructive pulmonary disease (COPD)	16
Chronic kidney disease (CKD)	12
Diabetes mellitus (NIDDM)	10
Diabetes mellitus (IDDM)	12
Chronic liver diseases	7
History of stroke	6
Elective surgery	30
Emergency surgery	52

Site of infection, %	
Lung	55
Abdomen	25
Bone or soft tissue	5
Surgical wound	2
Urogenital	2
Primary bacteremia	7
Other	4

Organ support, %	
Mechanical ventilation	84
Vasopressor therapy	60
Renal-replacement therapy	9

**Table 2:** Multivariate Cox regression analysis of 90-day mortality predictors.

**Variable**	**Hazard ratio**	**95% CI**	**p value**
Age > 65	1.43	0.97-2.11	0.0647
Gender, male	1.05	0.73-1.51	0.7681
BMI	0.97	0.94-1.00	0.0757
SOFA	1.10	1.02-1.18	0.0057
APACHE II	1.03	1.00-1.07	0.0229
Septic shock	0.84	0.52-1.37	0.5046
Arterial hypertension	1.35	0.93.1.98	0.1116
History of cancer	1.62	1.08-2.43	0.0182
COPD	0.98	0.64-1.51	0.9496
Chronic kidney disease	2.25	1.46-3.46	0.0002
Diabetes mellitus (IDDM)	1.62	0.99-2.64	0.0527
Diabetes mellitus (NIDDM)	1.65	0.96-2.83	0.0684
Coronary heart disease	0.89	0.48-1.66	0.7359
Chronic liver disease	0.61	0.29-1.30	0.2093
History of stroke	0.75	0.36-1.57	0.4582
Gram-positive infection	0.98	0.61-1.58	0.9635
Gram-negative infection	0.94	0.65-1.36	0.7755
Fungal infection	0.80	0.55-1.16	0.2509
Elective surgery	0.90	0.55-1.45	0.6725
Emergency surgery	0.80	0.51-1.26	0.3466

**Table 3:** Severity of disease with regard to chronic kidney disease.

	**All (n** = **482)**	**CKD (n** = **56)**	**no CKD (n** = **426)**	**P value**
SOFA	6.8 ± 3.6	8.9 ± 4.0	6.5 ± 3.4	< 0.0001
SOFA-Respiratory score	1.9 ± 0.8	2.0 ± 0.8	1.9 ± 0.8	0.1972
SOFA-Cardiovascular score	1.5 ± 1.0	1.9 ± 1.1	1.4 ± 0.9	0.0012
SOFA-Central Nervous System score	1.9 ± 1.1	2.0 ± 1.1	1.9 ± 1.0	0.3843
SOFA-Renal score	0.8 ± 1.2	1.8 ± 1.4	0.7 ± 1.0	< 0.0001
SOFA-Coagulation score	0.3 ± 0.6	0.6 ± 0.8	0.3 ± 0.5	0.0242
SOFA-Hepatic score	0.4 ± 0.7	0.6 ± 0.8	0.4 ± 0.7	0.0599

Mortality analysis, %				
Death at day 28	19	43	15	< 0.0001
Death at day 90	30	54	27	< 0.0001
Length of stay in ICU (days)		17.6 ± 13.0	14.2 ± 15.0	0.0278

Organ support-free days:				
Ventilator-free (days)		4 ± 5	5 ± 4	0.1574
Vasopressor-free (days)		7 ± 6	11 ± 7	0.0030
Dialysis-free (days)		11 ± 8	14 ± 8	0.0002

Inflammatory values				
Leucocytes (1000/μl)		15 ± 6	13 ± 5	0.1162
CRP (mg/l) (n)		156 ± 76 (29)	152 ± 87 (201)	0.6213
Procalcitonin (ng/dl) (n)		5.1 ± 7.3 (47)	4.4 ± 11.3 (374)	0.0061

Kidney and liver values				
Urine output (ml/d)		2161 ± 1767	3175 ± 1295	<0.0001
Urine output (ml/kg/h)		1.0 ± 0.9	1.7 ± 0.8	<0.0001
Creatinine (mg/dl)		2.1 ± 1.2	1.1 ± 0.9	<0.0001
Creatinine clearance (ml/min)		55 ± 37	113 ± 72	<0.0001
Alanine aminotransferase (IU/l)		76 ± 152	105 ± 217	0.0171
Aspartate aminotransferase (IU/l)		181 ± 331	196 ± 712	0.6239
Bilirubin (mg/dl)		1.4 ± 1.6	1.2 ± 2.1	0.2377

Additional laboratory values				
Lactate (mmol/l)		2.2 ± 1.7	1.6 ± 0.9	0.0019
Base excess (mmol/l)		0.6 ± 5.3	3.0 ± 4.0	0.0014
Platelets (1000/μl)		248 ± 162	315 ± 156	0.0004
Hematocrit (%)		27.4 ± 2.7	27.7 ± 3.6	0.8515
